# Selective and Continuous Transarterial Heparin Infusion: Postmicrosurgical Therapy of Lower Leg Reconstruction for Cases with Recipient Artery Damage

**DOI:** 10.29252/wjps.8.3.298

**Published:** 2019-09

**Authors:** Masayuki Okochi, Yuzo Komuro, Kazuki Ueda

**Affiliations:** 1 Department of Plastic and Reconstructive Surgery, Teikyo University, Tokyo, Japan;; 2 Department of Plastic and Reconstructive Surgery, Jusendo General Hospital, Tokyo, Japan

**Keywords:** Microsurgery, Postoperative therapy, Anticoagulant therapy, Free flap

## Abstract

**BACKGROUND:**

Microsurgical lower extremity reconstruction is challenging because of high incidence of vascular thrombosis compared to microsurgical head and neck reconstruction. The risk of vascular pedicle thrombosis increases, if patients have arterial sclerosis or intimal dissection at the recipient artery. We performed selective and continuous transarterial heparin infusion for postoperative anticoagulant therapy.

**METHODS:**

Fifteen patients (10 men and 5 women; mean age of 55.1 years; range of 16–86 years) received lower leg reconstruction using free flap. Postoperatively, a catheter was inserted into the femoral artery during surgery. Heparin infusion was performed through the catheter as a postoperative therapy for patients who had a risk factor of vascular pedicle thrombosis. Until two days post-operation, heparin was started between 5,000 and 10,000 IU per day. In postoperative days 3 and 4, half of the initial dose of heparin was administered. In postoperative days 5 and 6, 25% of the initial dose of heparin was administered.

**RESULTS:**

Recipient arteries were the posterior tibial (n=11), anterior tibial (n=2), lateral circumflex femoral (n=1), and medial sural (n=1) arteries. Thirteen of the 15 cases showed arterial sclerosis or intimal dissection at the recipient artery. There was no case of vascular thrombosis. Hematoma formation at flap recipient was observed in four cases. Their initial heparin dose was than 8.5±1.7 U/kg/h.

**CONCLUSION:**

Continuous transarterial heparin infusion was an effective anticoagulant therapy for the patients who had received free tissue transfer to a lower extremity. The initial dose of heparin should not exceed 6.5 U/kg/h.

## INTRODUCTION

Many authors have reported successful reconstruction using the free flap procedure.^1^^-^^29^ The frequency of vascular pedicle thrombosis after free tissue transfer for head and neck region is less than 5%.^2^^–^^8^ On the other hand, the success rate of lower leg reconstruction using flap is still low.^1^^,^^23^^–^^27^ Plastic surgeons often perform free tissue transfer to the lower extremities and vascular anastomosis for patients with arterial sclerosis. To avoid vascular pedicle thrombosis, we administered anticoagulant therapy for patients who had arterial sclerosis and received lower extremity reconstruction using free flap. 

Vascular thrombosis after free tissue transfer may lead to prolonged hospitalization or delays to starting postoperative rehabilitation. There is no guideline for postoperative therapy for patients who have had arterial sclerosis and received a free tissue transfer for a lower extremity. Heparin infusion is a well-known anticoagulant therapy and some authors reported the effectiveness of heparin infusion as an anticoagulant therapy after free tissue transfer.^12^^,^^13^^–^^17^^,^^20^^–^^22^

To prevent vascular pedicle compromise in patients whose recipient artery showed arterial sclerosis, we performed continuous and selective transarterial heparin infusion. We were able to prevent postoperative vascular pedicle thrombosis, even where patients showed arterial sclerosis in the lower leg. This report describes the details of our postoperative anticoagulant therapy for patients who had arterial sclerosis or malformation of the recipient artery. We also analyzed the relationship between amount of heparin and bleeding or thrombotic event at the surgical site. 

## MATERIALS AND METHODS

We retrospectively analyzed the patients’ medical records. From April 2008 through December 2018, fifteen patients (10 men and 5 women; mean age=55.1 years; range=16–86 years) received lower leg reconstruction using free flap, with continuous and selective transarterial heparin infusion as a postoperative anticoagulant therapy (Table 1). All surgeries were performed under general anesthesia. After preparation or debridement of the recipient site, flap was harvested and then fixed to the recipient site. Vascular anastomosis was performed under a microscope. 

**Table 1:** Patients profile

**No**	**Age, Sex**	**Location**	**Cause**	**Reconstruction**	**Recipient**	**Condition of Recipient Artery**			**Initial dose of heparin (U)**
						**Vascular malformation**	**Arterial sclerosis**	**Intimal dissection**	
1	60 M	Thigh	Osteomyelitis	LDMC	LCFA			(+)	8000
2	80 M	Knee	SCC	ALT	MSA		(+)		8000
3	16 M	Lower leg	Ischemia	LDMC	PTA			(+)	10000
4	47 F	Lower leg	Osteomyelitis	Fibula	ATA		(+)		10000
5	69 F	Achilles Tendon	Chronic ulcer	ALT	PTA		(+)	(+)	8000
6	69 M	Foot	DM	ALT	PTA			(+)	8000
7	65 M	Foot	DM	ALT	PTA		(+)	(+)	10000
8	45 M	Foot	SCC	DIEP	PTA			(+)	10000
9	59 F	Foot	AVM	ALT	PTA	(+)			5000
10	86 M	Foot	Burn	ALT	PTA		(+)		8000
11	41 M	Foot	BCC	ALT	PTA			(+)	8000
12	60 M	Achilles Tendon	Chronic ulcer	ALT	PTA	(+)			10000
13	26 F	Foot	AVM	ALT	ATA			(+)	8000
14	54 F	Foot	BCC	ALT	PTA		(+)	(+)	8000
15	50 M	Foot	DM	ALT	PTA		(+)		8000

M: Male, F: Female, LDMC: Latissimus dorsi myocutaneous flap, LCFA: Lateral circumflex femoral artery, SCC: Squamous cell carcinoma, ALT: Anterior lateral thigh flap, MSA: Medial sural artery, PTA: Posterior tibial artery, DM: Diabetes Mellitus, DIEP: Diep Inferior Epigastric artery, AVM: Arterial-venous malformation, BCC: Basal Cell Carcinoma

**Fig. 1:**
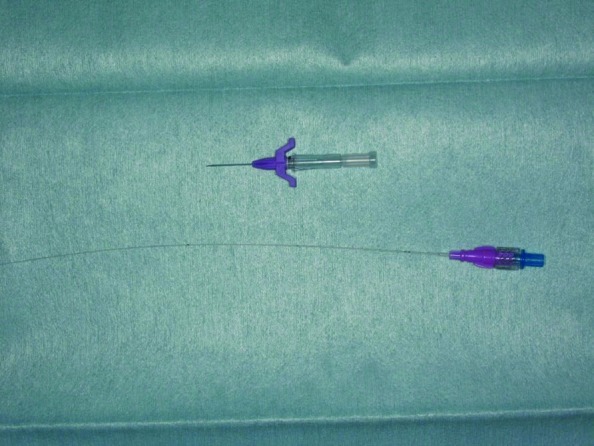
Catheter for continuous transarterial heparin infusion

**Fig. 2:**
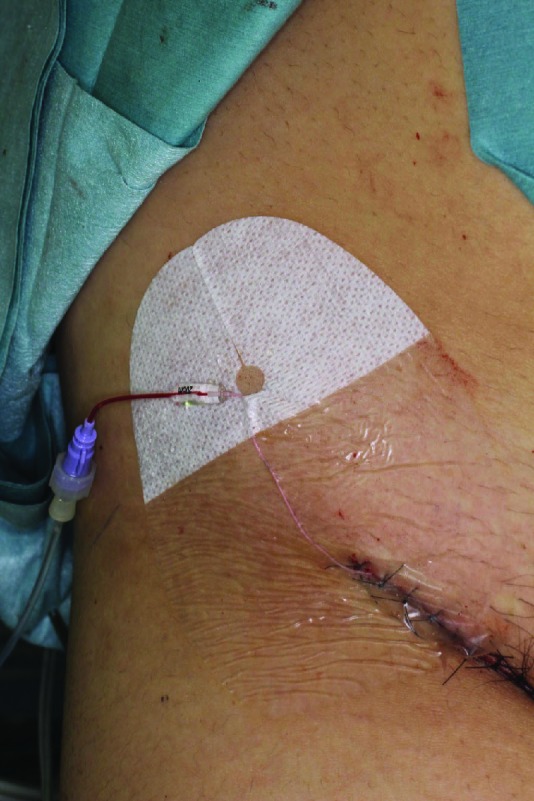
Catheter was introduced from the incision of femoral region

After vascular anastomosis, an incision was made at the femoral region of the affected site. From this incision, a 28G Argyle PI catheter (Covidien, Tokyo, Japan, Figure 1) was inserted into the femoral artery. The tip of catheter was sent to the 10-15cm distal site. Femoral incision was closed using 4-0 Vicryl (Ethicon, Somerville, New Jersey), 4-0 PDS (Ethicon), and 5-0 Ethilon (Ethicon). A catheter was introduced into this wound (Figure 2). Postoperatively, heparin infusion was performed through the catheter.

**Fig. 3:**
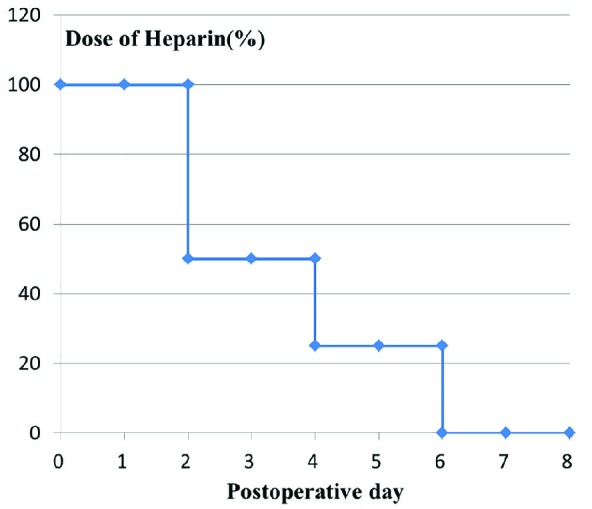
Relationship between dose of heparin and postoperative day

On the morning of day 2 post-operation, the daily dose of heparin was started at between 5,000 and 10,000 IU. In each of postoperative days 2 and 3, half of the initial dose of heparin was administered. In postoperative days 4 and 5, 25% of the initial dose heparin was administered. In postoperative day 6, heparin infusion was stopped. In postoperative day 8, the catheter was removed (Table 2, Figure 3). The activated partial thromboplastin time (APTT) level was checked every 24 hours until postoperative day seven. Flap survival was evaluated 8 days post-operation. Postoperatively, patients who had bleeding and needed additional treatment (opening of wound or ligation of bleeding vessel) were categorized as severe bleeding complication. 

**Table 2:** Protocol of transarterial heparin infusion

**Variable**	**Bleeding (n=5)**	**Non bleeding (n=10)**	***p***
APTT			
Preoperation	28.4±3.4	30.2±8.5	0.149
Postoperation	25.8±1.4	28.8±5.3	0.859
Initial dose heparin (U/kg/h)	8.5±1.7	5.9±0.56	0.0348

For our patients, differences between preoperative and postoperative APTT were analyzed with the Mann–Whitney U test. Differences in the initial dose of bone heparin between the complication and non-complication groups were also analyzed with the Mann–Whitney U test. The ratios of the postoperative bleeding at flap recipient site between high initial heparin dose (≥6.5 U/kg/h) group and low initial heparin dose (≤6.4 U/kg/h) group were evaluated using Fisher’s exact test. All calculations were performed using statistical software (SPSS, IBM Japan, Tokyo, Japan), and *p*<0.05 was considered significant.

## RESULTS

The causes of lower extremity reconstruction were Diabetes Mellitus foot (n=3), osteomyelitis (n=2), malignant tumor (n=2), Achilles tendon and soft tissue composite defect (n=2), vascular malformation (n=2), ulcer due to artery obstruction (n=1), scar contracture (n=1), and burn (n=1). The risk factors for vascular pedicle compromise were arteriosclerosis at the recipient site (n=7), intimal dissection (n=9), and vascular malformation (n=2). Flaps used were anterolateral thigh flap (n=11), fibula osteocutaneous flap (n=1), latissimus dorsi myocutaneous flap (n=1), and deep inferior epigastric artery perforator flap (n=1). 

Recipient sites were posterior tibial artery (n=1 1), anterior tibial artery (n=2), lateral circumflex femoral artery (n=1), and medial sural artery (n=1). The initial doses of heparin were 10,000 U (n=5), 8,000 U (n=9), and 5,000 U (n=1) (Table 1). The average initial dose of heparin was 6.4±1.8 U/kg/day. Postoperatively, no flap necrosis was observed. On the other hand, severe bleeding from flap recipient site was observed in four cases at 1 day post-operation. The average preoperative APTT value and highest postoperative APTT value were 27.5±10.5 and 27.9±4.6, respectively. There was no significant difference. 

In bleeding patients, preoperative and highest postoperative APTT values were 28.4±3.4 and 25.8±1.4, respectively. In non-bleeding patients, preoperative and highest postoperative APTT value was 30.2±8.5 and 28.8±5.3. There was no significant difference between pre- and post- operative APTT values in either bleeding patients (*p*=0.149) or non-bleeding patients (*p*=0.859). The average initial heparin dose among patients who had severe postoperative bleeding was 8.5±1.7/U/kg/h. 

On the other hand, the average initial heparin dose among non-bleeding patients was 5.9±0.56 U/kg/h. The initial dose of heparin per body weight was significantly higher than bleeding patients (*p*=0.035, Table 3). We divided our patients into two groups (HD; initial dose heparin ≥6.50 U/kg/h, LD; initial dose heparin ≤6.49 U/kg/h). In the HD group, three of the four patients had a bleeding complication. On the other hand, in the LD group, one of the nine patients had a bleeding complication. The rate of postoperative bleeding in the HD group was significantly higher than that of the LD group (*p*=0.041, Table 3).

**Table 3:** Relationship between bleeding complication and APTT value and dose of heparin

**Variable**	**Bleeding (n=5)**	**Non bleeding (n=10)**	***p***
LD	1	9	0.017
HD	4	1	

HD; initial dose heparin 6.50 U/kg/h, LD; initial dose heparin 6.49 U/kg/h)

## DISCUSSION

Free flap is a useful surgical procedure for the reconstruction of wide and complex tissue defects. In cases of vascular pedicle thrombosis, re-exploration is required. Re-exploration may lead to prolonged hospitalization. Various anticoagulant therapies after microsurgery have been reported.^8^^-^^20^ Heparin is one of the most popular drugs for anticoagulant therapy after microsurgery.^12^^,^^15^^,^^20^ To perform effective post-anticoagulant therapy, high-concentration anticoagulant drugs should be delivered for vascular pedicle. Ashjian et al. compared effectiveness between 325 mg/day of aspirin and 5000 U/day heparin infusion.^14^

They found no significant differences between effectiveness of aspirin and heparin. Khouri et al. examined 493 free flap transfers and compared effectiveness among various postoperative anticoagulant therapies.^1^ They concluded that only postoperative subcutaneous heparin injection could decrease rate of flap failure. Some authors reported that post-microsurgical anticoagulant therapy does not decrease the risk of vascular pedicle compromise. However, in their report, most of their cases were head and neck reconstruction cases.^8^^,^^9^

The rate of free flap failure among patients who underwent head and neck reconstruction using free flap was only 0–5%, even without anticoagulant therapy after microsurgery.^2^^–^^7^ These results suggest that it is very difficult to further decrease risk of free flap failure. However, the rate of free flap failure after lower leg reconstruction remains high.^23–27^ Intraoperatively, we sometimes experience cases who have recipient artery with arterial sclerosis or dissection of intima. 

We felt the need for post-microsurgical anticoagulant therapy in these cases. We used heparin via catheter inserted into the femoral artery. From the survey by Xipoleas et al., two of 102 microsurgeons performed systemic venous 5000 U/day heparin infusion until 1 day after free tissue transfer to the lower extremity.^26^ Eley et al. also performed 2,500–10,000 U/day systemic low molecular weight heparin infusion after free flap transfer.^11^ From these results, using heparin 10,000 U/day causes less than 2.3% hematoma formation. 

We believe this dose of heparin is not effective. Based on the 2014 European Society of Cardiology guideline, more than 18 U/kg/h heparin is required to keep 1.5 to 2.3 times higher APTT value than normal.^30^ The average body weight of our patients was 55.1±18.7 kg. From the guideline, about 23,000 U/day heparin should be administered considering this average weight. However, if we use such high doses of systemic heparin infusion, postoperative hematoma or bleeding may cause. 

To send high-concentration heparin for vascular pedicle without hematoma formation or bleeding, some authors perform local transarterial heparin infusion. Fukui et al. reported 240,000 U of urokinase, 40 μg PGE1, low molecular weight dextran, and 10,000 U of heparin were administered until 10 days postoperatively.^28^ Saito et al. also performed transarterial heparin infusion. In their report, 2,000 U daily heparin with 40 μg PGE1 were administered until one week post-operation.^29^

On the other hand, we used an initial heparin dosage of 10,000–5,000 U. None of our cases showed increased APTT value. However, no vascular thrombosis was observed. Some authors reported that the most common reason for free flap failure after lower leg reconstruction was vein thrombosis.^22^^,^^31^ To send heparin to the anastomotic site of the vein, the catheter should be inserted into the flap vein. However, it is very difficult to insert catheter to flap vein. In our study, four cases showed bleeding from surgical site. This result suggests that we could send high-concentration heparin for vascular pedicle of flap without inserting catheter for flap vein. 

## CONFLICT OF INTEREST

The authors declare no conflict of interest.

## References

[1] Khouri RK, Cooley BC, Kunselman AR, Landis JR, Yeramian P, Ingram D, Natarajan N, Benes CO, Wallemark C (1998). A prospective study of microvascular free-flap surgery and outcome. Plast Reconstr Surg.

[2] Eckardt A, Fokas K (2003). Microsurgical reconstruction in the head and neck region: an 18-year experience with 500 consecutive cases. J Craniomaxillofac Surg.

[3] Fukuiwa T, Nishimoto K, Hayashi T, Kurono Y (2008). Venous thrombosis after microvascular free-tissue transfer in head and neck cancer reconstruction. Auris Nasus Larynx.

[4] Nakamizo M, Yokoshima K, Yagi T (2004). Use of free flaps for reconstruction in head and neck surgery: a retrospective study of 182 cases. Auris Nasus Larynx.

[5] Pohlenz P, Blessmann M, Heiland M, Blake F, Schmelzle R, Li L (2007). Postoperative complications in 202 cases of microvascular head and neck reconstruction. J Craniomaxillofac Surg.

[6] Chalian AA, Anderson TD, Weinstein GS, Weber RS (2001). Internal jugular vein versus external jugular vein anastamosis: implications for successful free tissue transfer. Head Neck.

[7] Chernichenko N, Ross DA, Shin J, Chow JY, Sasaki CT, Ariyan S (2008). Arterial coupling for microvascular free tissue transfer. Otolaryngol Head Neck Surg.

[8] Reiter M, Kapsreiter M, Betz CS, Harreus U (2012). Perioperative management of antithrombotic medication in head and neck reconstruction-a retrospective analysis of 137 patients. Am J Otolaryngol.

[9] Parra L, Andres J, Robustillo M, Garcia C, Iglesias I, Diaz A (2018). Ipsilateral Arteriovenous Loop and Latissimus Dorsi Free Flap for Knee Reconstruction in an Elderly Patient: A Case Report. World J Plast Surg.

[10] Khoshnevis J, Dashti T, Ebrahimi M, Azargashb E, Kalantar Motamedi M (2018). Anastomosis of Free Flap Pedicle to Great Vessels. World J Plast Surg.

[11] Buono P, Castus P, Dubois-Ferriere V, Ruegg EM, Uckay I, Assal M, Pittet-Cuenod B, Modarressi A (2018). Muscular Versus Non-Muscular Free Flaps for Soft Tissue Coverage of Chronic Tibial Osteomyelitis. World J Plast Surg.

[12] Numajiri T, Sowa Y, Nishino K, Arai A, Tsujikawa T, Ikebuchi K, Nakano H, Sakaguchi H (2016). Use of systemic low-dose unfractionated heparin in microvascular head and neck reconstruction: Influence in free-flap outcomes. J Plast Surg Hand Surg.

[13] Blackburn TK, Java KR, Lowe D, Brown JS, Rogers SN (2012). Safety of a regimen for thromboprophylaxis in head and neck cancer microvascular reconstructive surgery: non-concurrent cohort study. Br J Oral Maxillofac Surg.

[14] Ashjian P, Chen CM, Pusic A, Disa JJ, Cordeiro PG, Mehrara BJ (2007). The effect of postoperative anticoagulation on microvascular thrombosis. Ann Plast Surg.

[15] Chen CM, Ashjian P, Disa JJ, Cordeiro PG, Pusic AL, Mehrara BJ (2008). Is the use of intraoperative heparin safe?. Plast Reconstr Surg.

[16] Sigaux N, Philouze P, Boucher F, Jacquemart M, Frobert P, Breton P (2017). Efficacy of the postoperative management after microsurgical free tissue transfer. J Stomatol Oral Maxillofac Surg.

[17] Senchenkov A, Lemaine V, Tran NV (2015). Management of perioperative microvascular thrombotic complications - The use of multiagent anticoagulation algorithm in 395 consecutive free flaps. J Plast Reconstr Aesthet Surg.

[18] Weinand C, Dittes C (2019). Soft Tissue Mandibula and Tongue Reconstruction Using A Suprafascial, Folded, Deepithelialized Antero-Lateral Thigh Perforator Free Flap. World J Plast Surg.

[19] Gholami M, Hedjazi A, Kiamarz Milani A (2019). Evaluation of Anatomic Variations of Fibula Free Flap in Human Fresh Cadavers. World J Plast Surg.

[20] Andresen DM, Barker JH, Hjortdal VE (2002). Local heparin is superior to systemic heparin in preventing arterial thrombosis. Microsurgery.

[21] Hidalgo DA, Disa JJ, Cordeiro PG, Hu QY (1998). A review of 716 consecutive free flaps for oncologic surgical defects: refinement in donor-site selection and technique. Plast Reconstr Surg.

[22] Pugh CM, Dennis RH, 2nd, Massac EA (1996). Evaluation of intraoperative anticoagulants in microvascular free-flap surgery. J Natl Med Assoc.

[23] Trost O, Kadlub N, Malka G, Trouilloud P, Danino AM (2006). Microvascular free flap reconstruction in pediatric lower extremity trauma. Plast Reconstr Surg.

[24] Rinker B, Valerio IL, Stewart DH, Pu LL, Vasconez HC (2005). Microvascular free flap reconstruction in pediatric lower extremity trauma: a 10-year review. Plast Reconstr Surg.

[25] Lee KT, Jeon BJ, Lim SY, Pyon JK, Bang SI, Oh KS, Mun GH (2012). The effects of ketorolac on microvascular thrombosis in lower extremity reconstruction. Plast Reconstr Surg.

[26] Xipoleas G, Levine E, Silver L, Koch RM, Taub PJ (2007). A survey of microvascular protocols for lower-extremity free tissue transfer I: perioperative anticoagulation. Ann Plast Surg.

[27] Xipoleas G, Levine E, Silver L, Koch RM, Taub PJ (2008). A survey of microvascular protocols for lower extremity free tissue transfer II: postoperative care. Ann Plast Surg.

[28] Fukui A, Maeda M, Mine T, Inada Y, Mizumoto S, Tamai S (1992). Continuous local intraarterial infusion after prolonged arterial stasis in the fingers and toes. Microsurgery.

[29] Saito A, Sawaizumi M, Imai T, Matsumoto S (2010). Continuous local intraarterial infusion of anticoagulants for microvascular free tissue transfer in primary reconstruction of the lower limb following resection of sarcoma. Microsurgery.

[30] Konstantinides S, Torbicki A, Agnelli G, Danchin N, Fitzmaurice D, Galie N, Gibbs J, Huisman M, Humbert M, Kucher N (2014). Task force for the diagnosis and management of acute pulmonary embolism of the European Society of Cardiology (ESC). Eur Heart J.

[31] Culliford ATt, Spector J, Blank A, Karp NS, Kasabian A, Levine JP (2007). The fate of lower extremities with failed free flaps: a single institution’s experience over 25 years. Ann Plast Surg.

